# The Immunosuppressive Roles of PD-L1 during Influenza A Virus Infection

**DOI:** 10.3390/ijms24108586

**Published:** 2023-05-11

**Authors:** Hongya Ning, Shih-Hsin Chiu, Xiaodong Xu, Yanmei Ma, Ji-Long Chen, Guihong Yang

**Affiliations:** Key Laboratory of Animal Pathogen Infection and Immunology of Fujian Province, College of Animal Sciences (College of Bee Science), Fujian Agricultural and Forestry University, Fuzhou 350002, Chinachenjilong@tom.com (J.-L.C.)

**Keywords:** programmed death ligand 1, influenza A virus H1N1 subtype, SHP2, inflammation, virus replication, cytokines

## Abstract

The clinical benefits of targeting programmed death-ligand 1 (PD-L1) in various cancers represent a strategy for the treatment of immunosuppressive diseases. Here, it was demonstrated that the expression levels of PD-L1 in cells were greatly upregulated in response to H1N1 influenza A virus (IAV) infection. Overexpression of PD-L1 promoted viral replication and downregulated type-I and type-III interferons and interferon-stimulated genes. Moreover, the association between PD-L1 and Src homology region-2, containing protein tyrosine phosphatase (SHP2), during IAV/H1N1 infection was analyzed by employing the SHP2 inhibitor (SHP099), siSHP2, and pNL-SHP2. The results showed that the expressions of PD-L1 mRNA and protein were decreased under SHP099 or siSHP2 treatment, whereas the cells overexpressing SHP2 exhibited the opposite effects. Additionally, the effects of PD-L1 on the expression of p-ERK and p-SHP2 were investigated in PD-L1-overexpressed cells following WSN or PR8 infection, determining that the PD-L1 overexpression led to the decreased expression of p-SHP2 and p-ERK induced by WSN or PR8 infection. Taken together, these data reveal that PD-L1 could play an important role in immunosuppression during IAV/H1N1 infection; thus, it may serve as a promising therapeutic target for development of novel anti-IAV drugs.

## 1. Introduction

Influenza A virus (IAV) is a top global threat to public health, causing severe pneumonia. IAV infection motivates host innate immune responses, including expression of type-I and type-III interferons (IFNs), through manifold signal transduction pathways, such as pathogen recognition receptor-mediated signaling [1,2,3,4,5,6,7,8]. Upon the recognition of viral pathogen-associated molecular patterns (PAMPs) by pattern recognition receptors (PRRs), inflammatory cytokines, including interleukin-1β (IL-1β), IL-6, and tumor necrosis factor alpha, are produced in hosts in response to IAV infection [5,9,10,11,12,13,14]. Although these cytokines play critical antiviral roles in the first line of host defense against IAV infection, excessive inflammatory responses to IAV infection can lead to viral pathogenesis [9,12]. In the past decades, many strategies to achieve sustained viral replication have been observed, such as employing mitogen-activated protein kinase/extracellular signal regulated kinase (MAPK/ERK) and signal transducer and activation of transcription (STAT)-mediated signaling [15,16,17,18,19,20,21,22,23]. However, the mechanisms underlying the immunosuppressive activities of IAV remain poorly understood.

Immune evasion is a survival strategy for cancers, taking advantage of molecular signaling such as by upregulating the programmed death ligand 1 (PD-L1) and Src homology region-2, containing protein tyrosine phosphatase (SHP2). PD-L1 is a type-I transmembrane protein and is also known as B7-H1 or CD274 [24]. It consists of an extracellular domain (ECD), a transmembrane domain (TMD), and a cytoplasmic domain (CD) [20,21,25]. As an important immune checkpoint, high expression of PD-L1 is detected in various immune cells and cancer cells, and PD-L1 can be transported into the nucleus and then participates in regulating gene transcription, which is involved in interferon signaling [18,26], NF-kB signaling [19,27,28], immune checkpoints [19,22,23,29,30,31], and inflammation [31,32,33]. In the context of cancers, PD-L1 binding to PD-1 on tumor-specific cytotoxic T lymphocytes (CTLs) can recruit SHP2 to the C-terminal of the PD-1 intracellular domain, leading, subsequently, to the inhibition of downstream PI3K/Akt signaling, which would downregulate cell survival gene BCL-XL expression and result in the dysfunction or apoptosis of T cells [21,34,35,36]. Antagonizing the PD-L1/PD-1 interaction can revert the exhausted phenotype of T cells and allows for the efficient killing of cancer cells [22,23,37,38,39,40]. These phenomena demonstrate the importance of PD-L1 signaling in cancer progression, but the immunosuppressive potential of PD-L1 in response to viral infection is still not clear, especially IAV. Recently, Wang et al. have found that SHP2 not only enables to induce ERK phosphorylation but also functions as a negative factor in the activation of p-STAT1 [35]. These results disclosed that SHP2 is crucial for the suppression of host antiviral response. Thus, SHP2 depletion may be an effective approach against IAV infection. Based on these observations, further studies are needed to investigate whether SHP2 can mediate the activities of PD-L1 in response to IAV infection. The brilliant success achieved by targeting PD-L1 in cancer treatment provides a promising route for the development of anti-IAV immunotherapy. However, few studies have exploited the potential of PD-L1 in immune evasion in response to IAV infection.

In this study, we aimed to explore the role of PD-L1 in host innate immune responses to IAV/H1N1 infection. Here, we found that PD-L1 was profusely expressed in IAV-infiltrating cells and played a critical role in limiting anti-IAV/H1N1 immunity. Furthermore, the expression of PD-L1 can be regulated by SHP2 activation. These findings reveal the regulation of PD-L1 during IAV infection and provide an extensive understanding of viral immune evasion.

## 2. Results

### 2.1. The Expression of PD-L1 Expression Is Up-Regulated in IAV/H1N1-Infected A549 Cells

**Figure 1:**
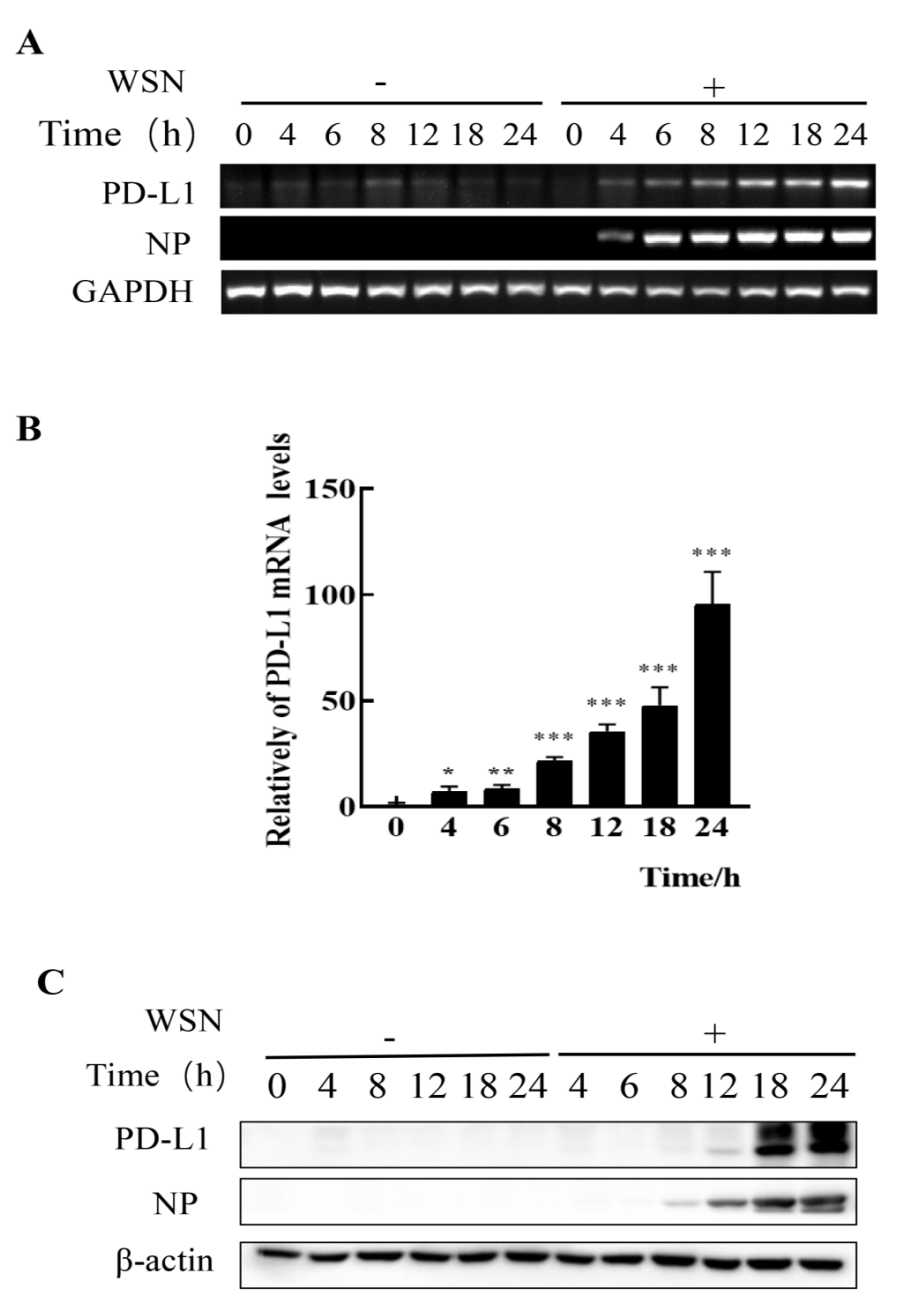
Robust expression of PD-L1 is induced by WSN infection. A549 cells were infected with or without WSN (MOI = 1) for 0, 4, 6, 8, 12, 18, and 24 h, and then the expression levels of *PD-L1* mRNA were determined by RT-PCR (**A**) and qRT-PCR (**B**). The expression levels of PD-L1 protein were examined by Western blotting (**C**). *GAPDH* and β-actin were used as internal standards. Three independent experiments were performed. * *p* < 0.05; ** *p* < 0.01; *** *p* < 0.001.

**Figure 2:**
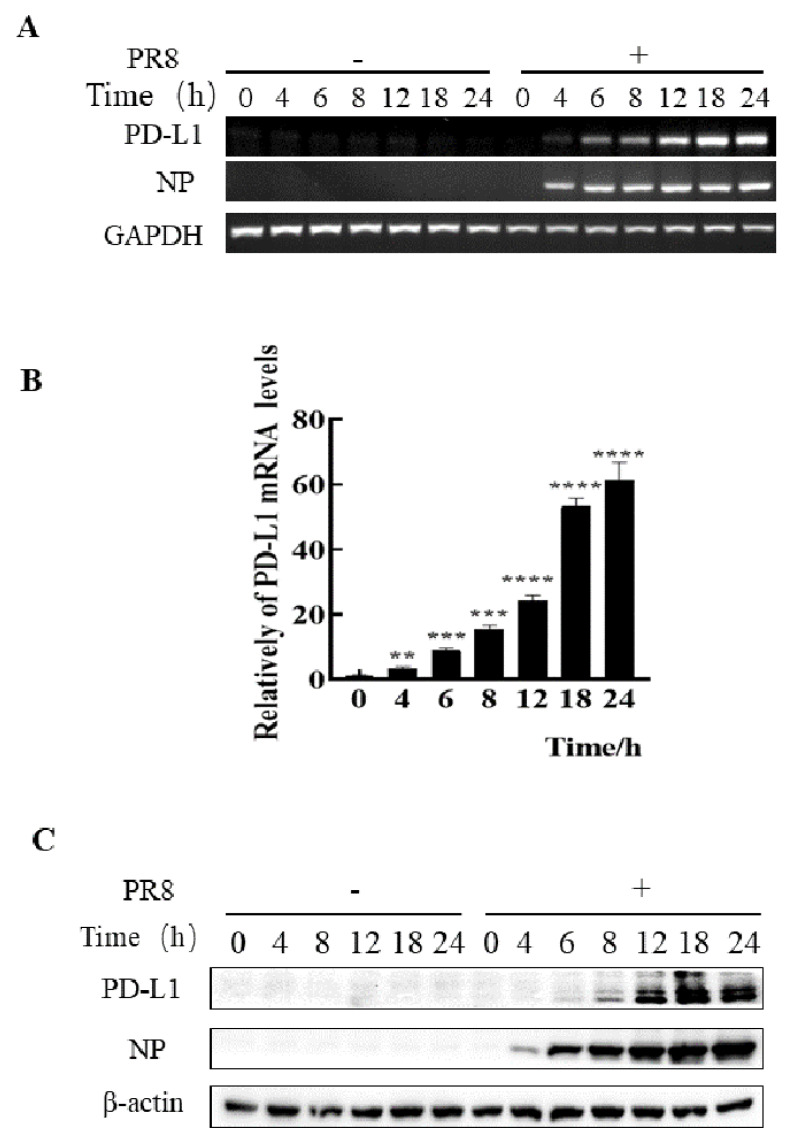
Robust expression of PD-L1 is induced by PR8 infection. A549 cells were infected with or without PR8 (MOI = 1) for 0, 4, 6, 8, 12, 18, and 24 h, and then the expression levels of *PD-L1* mRNA were determined by RT-PCR (**A**) and qRT-PCR (**B**). The expression levels of PD-L1 were examined by Western blotting (**C**). *GAPDH* and β-actin were used as internal standards. Three independent experiments were performed. ** *p* < 0.01; *** *p* < 0.001, **** *p* < 0.0001.

To assess whether IAV infiltrating non-immune cells can express PD-L1, we examined the expression levels of PD-L1 in A549 cells infected with IAV WSN and PR8. In comparison to the cells without WSN or PR8 infection, mRNA and protein levels of PD-L1 were profusely expressed in WSN- or PR8-infected A549 cells. Meanwhile, the viral NP expression was also increased (Figure 1 and Figure 2). Therefore, these results demonstrate that IAV infection can trigger the expression of PD-L1 in A549 cells.

### 2.2. Overexpression of PD-L1 Facilitates IAV/H1N1 Infection

**Figure 3:**
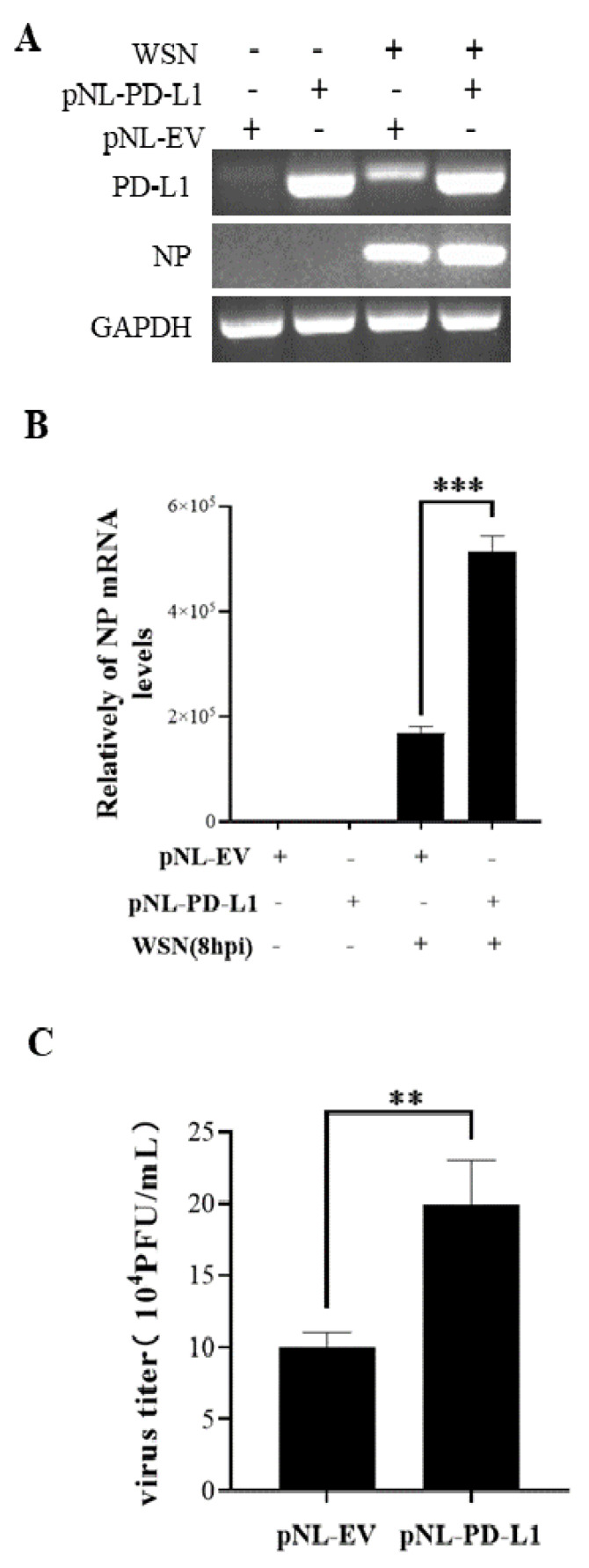
Overexpression of *PD-L1* induces *NP* expression and IAV replication. The expression levels of *PD-L1* and *NP* mRNA in A549 cells were detected by RT-PCR (**A**) and qRT-PCR (**B**) after infection with WSN (MOI = 1) at 8 h. After infection with or without WSN (MOI = 1) at 18 h, the supernatants were collected, and the virus titer was detected by plaque assay (**C**). *GAPDH* was used as an internal standard. Three independent experiments were performed for each incubation. ** *p* <0.01; *** *p* < 0.001.

**Figure 4:**
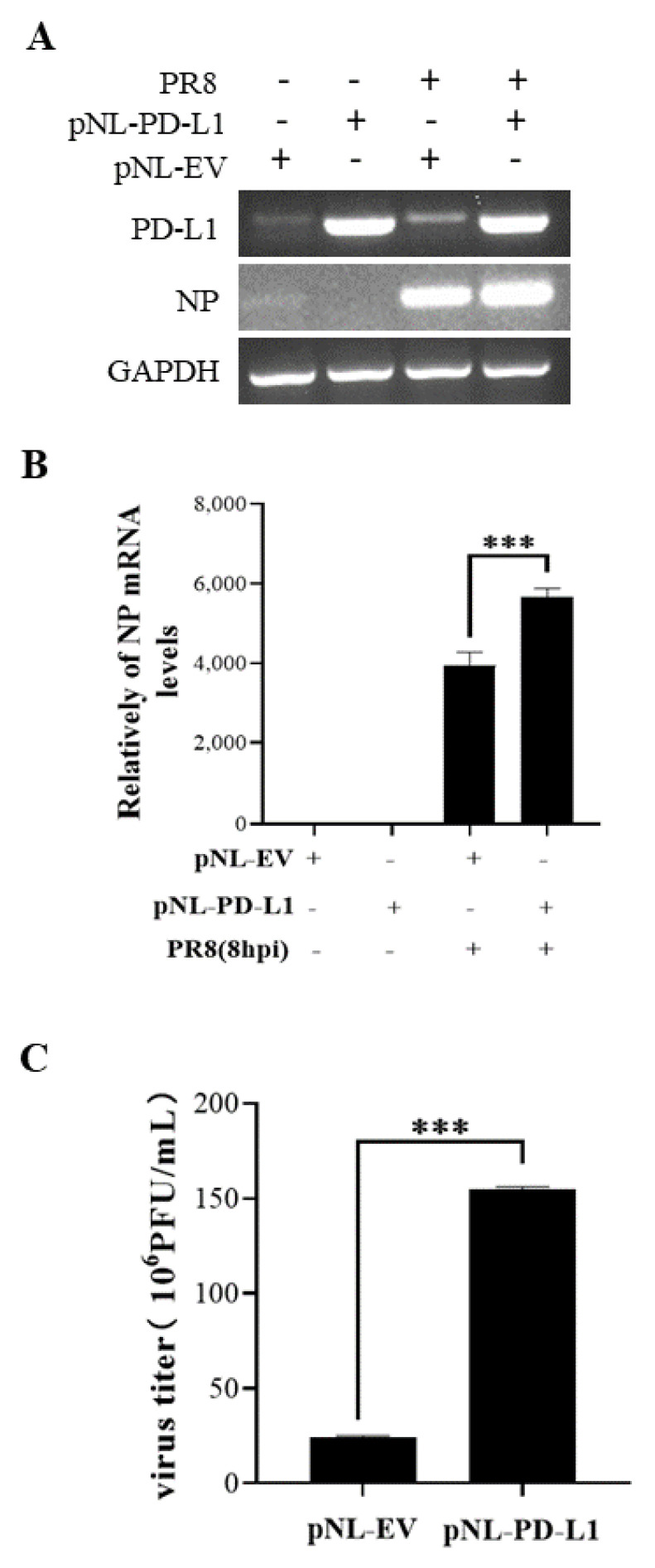
Overexpression of *PD-L1* induces *NP* mRNA expression and IAV replication. A549 cells were infected with PR8 (MOI = 1) for 8 h, and the mRNA levels of *PD-L1* and *NP* were measured by RT-PCR (**A**) and qRT-PCR (**B**). After infection with PR8 (MOI = 1) at 8 h, the supernatants were collected, and plaque assay was used to detect virus titers (**C**). *GAPDH* was used as an internal standard. Three independent experiments were performed for each incubation.*** *p* < 0.001.

To investigate the potential roles of PD-L1 in response to IAV/H1N1 infection, the effects of PD-L1 on the virus replication in A549 cells were analyzed. PD-L1-overexpressed cells were generated from A549 cells after transfection with pNL-PD-L1, and the control cells were transfected with pNL-EV. Then, the cells were infected with WSN. The mRNA levels of *PD-L1* and *NP* in A549 cells were examined using RT-PCR and qRT-PCR. As shown in Figure 3, the *PD-L1* overexpression caused a significant increase in *NP* expression, compared with the controls. Consistent with the change in the *NP* expression, the virus titer was significantly increased in cells overexpressing PD-L1. Meanwhile, the replication of PR8 in A549 cells was measured. Similarly, the PD-L1 overexpression significantly increased the expression of *NP* mRNA and the virus titer in A549 cells after virus infection (Figure 4). These results suggest that up-regulation of PD-L1 is required for IAV infection.

### 2.3. PD-L1 Inhibits Host Innate Immunity against IAV/H1N1

**Figure 5:**
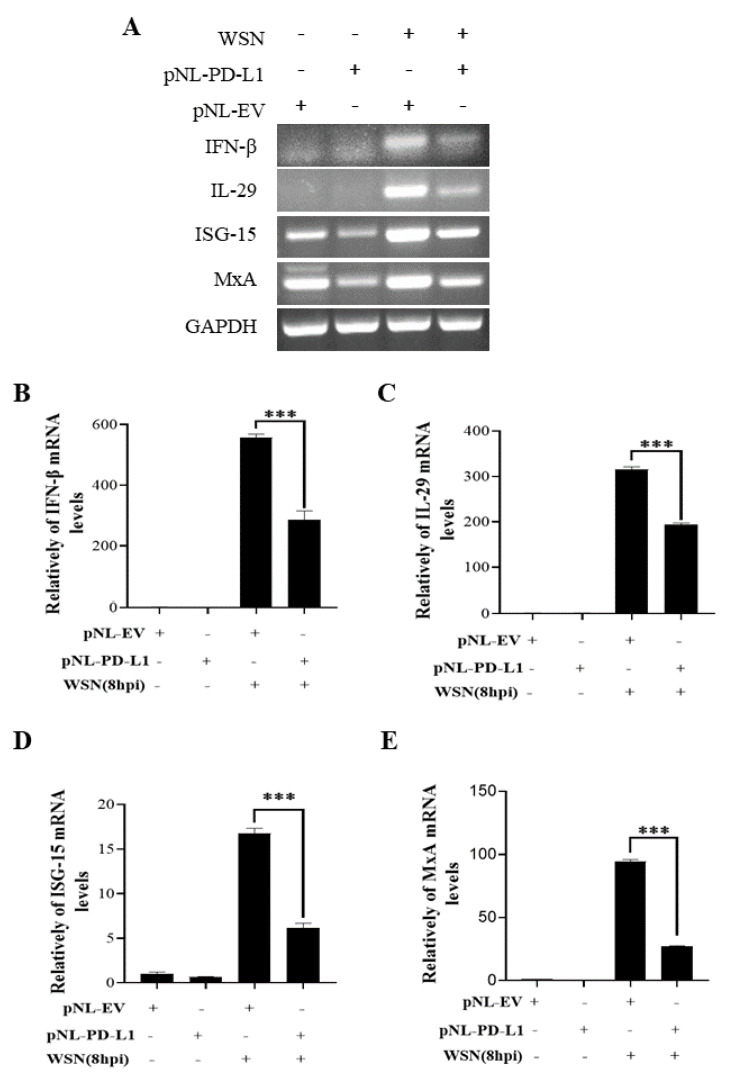
Overexpression of *PD-L1* regulates the expression of cytokines during WSN infection. A549 cells bearing pNL-PD-L1 or pNL-EV were infected with or without WSN (MOI = 1) for 8 h. The expression levels of *IFN-β*, *IL-29*, *ISG15*, and *MxA* mRNAs in these cells were examined by RT-PCR (**A**) and qRT-PCR (**B**–**E**). *GAPDH* was used as an internal standard. Three independent experiments were performed for each incubation. *** *p* < 0.001.

**Figure 6:**
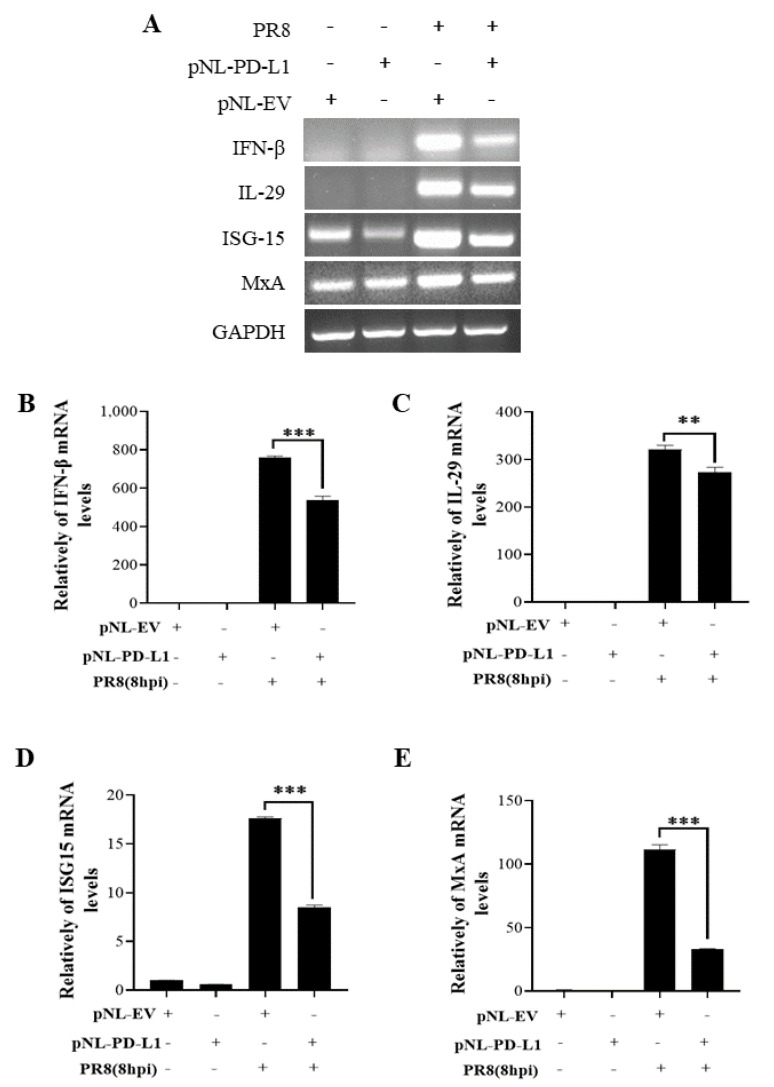
Overexpression of *PD-L1* regulates the expression of cytokines during PR8 infection. A549 cells bearing pNL-PD-L1 or pNL-EV were infected with or without PR8 (MOI = 1) for 8 h. The mRNA expression levels of *IFN-β*, *IL-29*, *ISG15*, and *MxA* in these cells were examined by RT-PCR (**A**) and qRT-PCR (**B**–**E**). *GAPDH* was used as an internal standard. Three independent experiments were performed for each incubation.** *p* < 0.01; *** *p* < 0.001.

To further explore the function of PD-L1 in suppressing host antiviral responses to IAV infection, we analyzed the expression levels of innate immunity-related genes, including *IFN-β*, *IL-29*, *ISG15*, and *MxA*, in cells overexpressing *PD-L1* during WSN or PR8 infection. The ectopic expression of *PD-L1* in A549 cells exhibited a significant down-regulation in WSN-induced *IFN-β, IL-29*, *ISG15*, and *MxA* expression, compared with the control cells (Figure 5). As expected, the similar diminished expression levels of *IFN-β*, *IL-29*, *ISG15*, and *MxA* were also observed in *PD-L1*-overexpressed cells upon PR8 infection (Figure 6). These results indicate that induction of *PD-L1* can suppress the host innate immunity by negatively regulating the expression of *type-I* and *type-III IFNs* and *ISGs*, thus facilitating IAV infection.

### 2.4. SHP2 Regulates PD-L1 Expression and Viral Replication during IAV/H1N1 Infection

**Figure 7:**
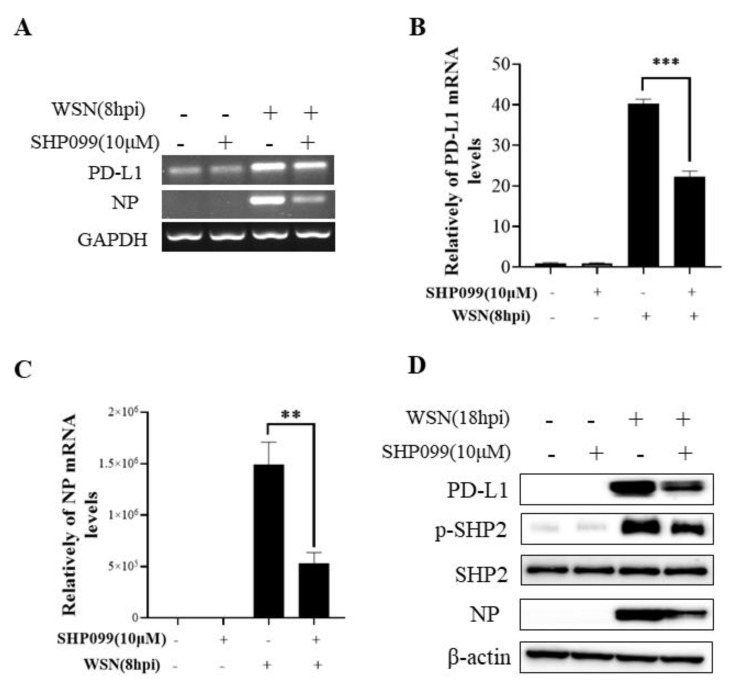
SHP2 regulates the expression of PD-L1 and NP in response to WSN infection. Probe detection in A549 cells was performed with SHP099, an allosteric small molecule inhibitor of SHP2, and the cells were infected with or without WSN (MOI = 1) for 8 h. Decreased mRNA levels of *PD-L1* and *NP* driven by SHP099 treatment were observed using RT-PCR(**A**) and qRT-PCR (**B**,**C**). Western blotting showed the decreased expression levels of PD-L1 and NP proteins after WSN (MOI = 1) infection at 18 h (**D**). *GAPDH* and β-actin were used as internal standards. Three independent experiments were performed for each incubation. ** *p* < 0.01; *** *p* < 0.001.

**Figure 8:**
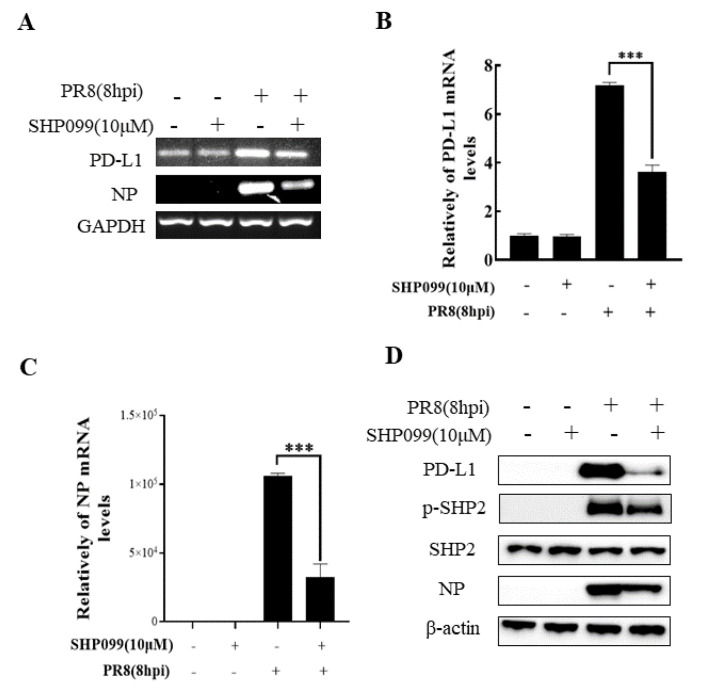
SHP2 regulates the expression PD-L1 and NP in response to PR8 infection. Probe detection in A549 cells was performed with SHP099, an allosteric small molecule inhibitor of SHP2, and the cells were infected with or without PR8 (MOI = 1) for 8 h. Decreased expression levels of *PD-L1* and *NP* mRNAs driven by SHP099 treatment were observed using RT-PCR (**A**) and qRT-PCR (**B**,**C**). Western blotting showed the decreased expression levels of PD-L1 and NP proteins after PR8 (MOI = 1) infection at 18 h (**D**). *GAPDH* and β-actin were used as internal standards. Three independent experiments were performed for each incubation.*** *p* < 0.001.

**Figure 9:**
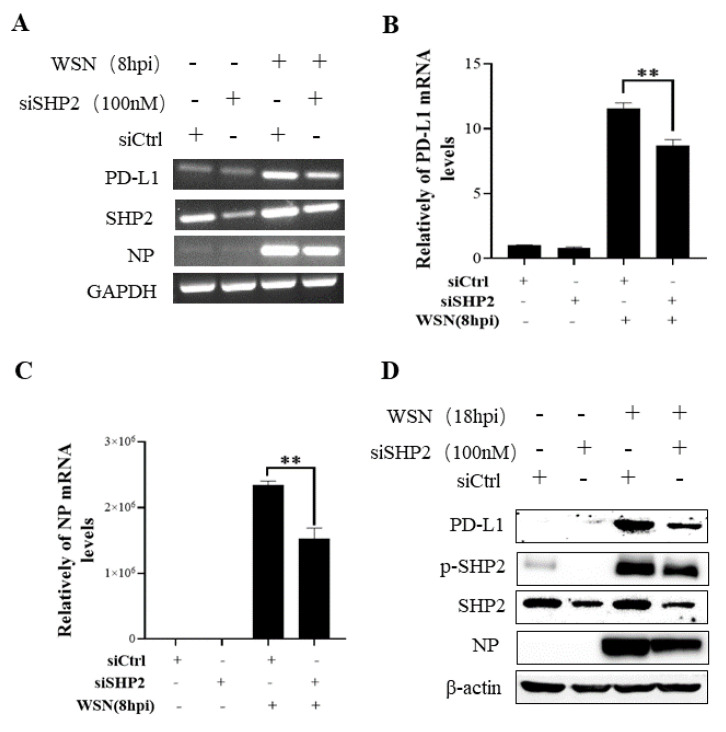
Targeted disruption of SHP2 inhibits PD-L1 and NP expression during WSN infection. A549 cells bearing siSHP2 or siCtrl were infected with or without WSN (MOI = 1) for 8 h. The decreased mRNA expression levels of *PD-L1* and *NP* in these cells were detected by RT-PCR (**A**) and qRT-PCR (**B**,**C**). With or without WSN (MOI = 1) infection for 18 h, Western blotting showed decreased expression levels of PD-L1 and NP protein, and decreased phosphorylation of SHP2 (**D**). *GAPDH* and β-actin were used as internal standards. Three independent experiments were performed for each incubation. ** *p* < 0.01.

**Figure 10:**
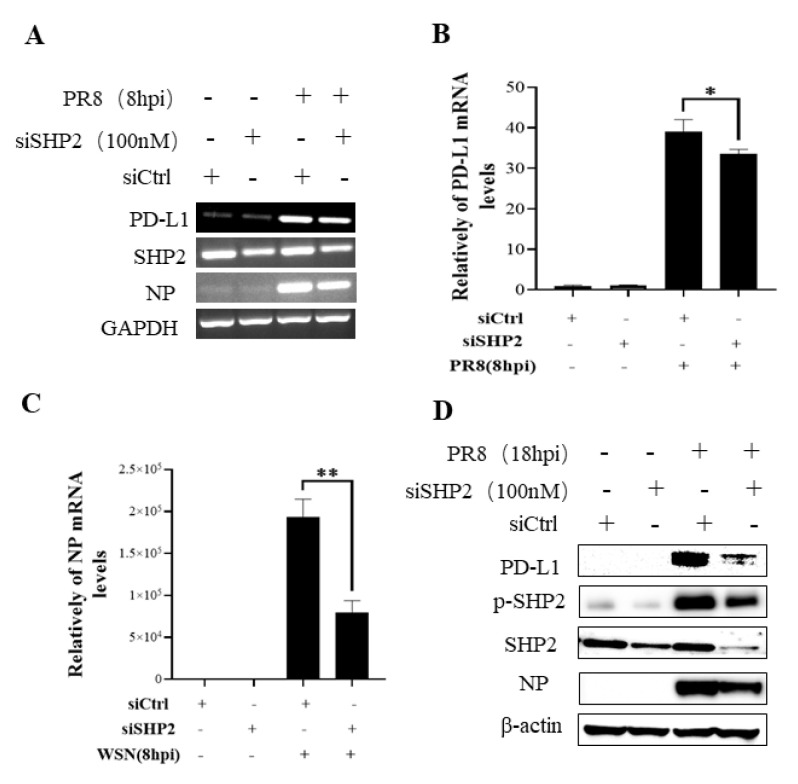
Targeted disruption of SHP2 inhibits PD-L1 and NP expression during PR8 infection. A549 cells bearing siSHP2 or siCtrl were infected with or without PR8 (MOI = 1) infection for 8 h. The decreased mRNA expression levels of *PD-L1* and *NP* in these cells were detected by RT-PCR (**A**) and qRT-PCR (**B**,**C**). With or without PR8 (MOI = 1) infection for 18 h, Western blotting showed decreased expression levels of PD-L1 and NP protein and decreased phosphorylation of SHP2 (**D**). *GAPDH* and β-actin were used as internal standard. Three independent experiments were performed for each incubation. * *p* < 0.05; ** *p* < 0.01.

**Figure 11:**
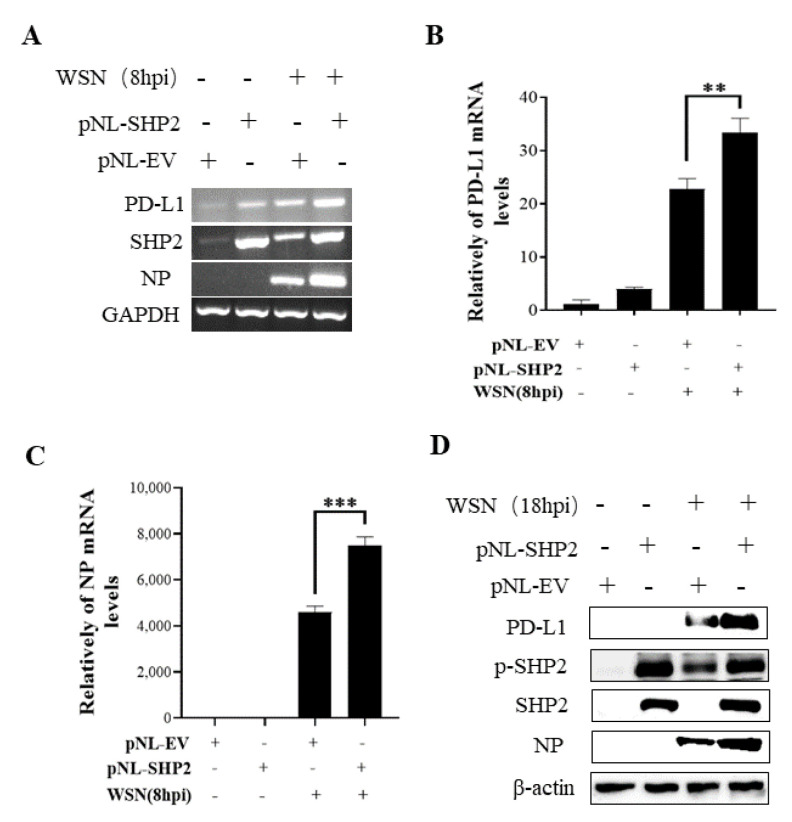
Overexpression of SHP2 promotes the expression of PD-L1 and NP during WSN infection. A549 cells were infected with or without WSN (MOI = 1) for 8 h after transfection of pNL-SHP2 or pNL-EV. Increased mRNA expression levels of *PD-L1* and *NP* in these cells were detected by RT-PCR (**A**) and qRT-PCR (**B**,**C**). With or without WSN (MOI = 1) infection for 18 h, Western blotting showed increased expression levels of PD-L1 and NP proteins (**D**). *GAPDH* and β-actin were used as internal standard. Three independent experiments were performed for each incubation. ** *p* < 0.01; *** *p* < 0.001.

**Figure 12:**
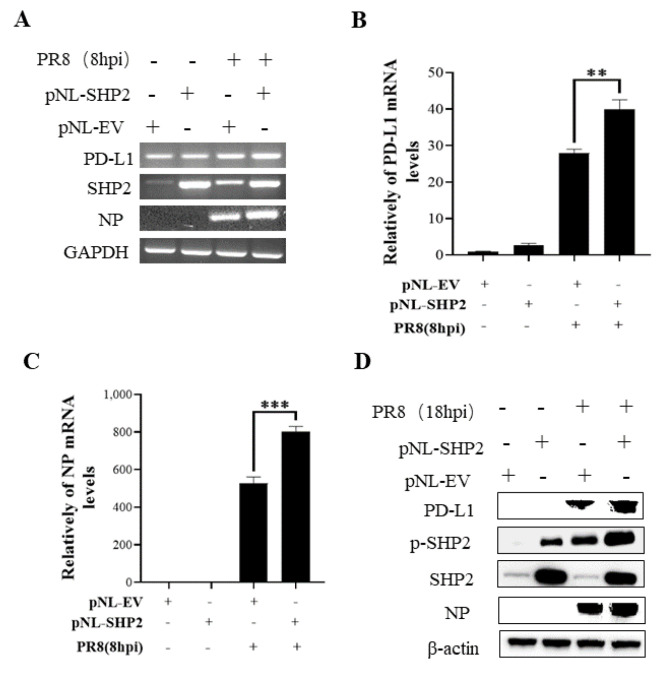
Overexpression of SHP2 promotes the expression of PD-L1 and NP during PR8 infection. A549 cells were infected with or without PR8 (MOI = 1) for 8 h after transfection of pNL-SHP2 or pNL-EV. Increased mRNA expression levels of *PD-L1* and *NP* in these cells were detected by RT-PCR (**A**) and qRT-PCR (**B**,**C**). With or without PR8 (MOI = 1) infection for 18 h, Western blotting showed the increased expression levels of PD-L1 and NP protein (**D**). *GAPDH* and β-actin were used as internal standard. Three independent experiments were performed for each incubation. ** *p* < 0.01; *** *p* < 0.001.

To understand the specific function of PD-L1 in response to IAV infection, it is important to analyze the association between PD-L1 and SHP2 upon WSN or PR8 challenge. The allosteric small molecular inhibitor of SHP2, SHP099, was employed to treat A549 cells prior to virus infection, and it was observed that the expressions of PD-L1 and NP were inhibited by SHP099 treatment (Figure 7 and Figure 8). Further, the A549 cells lacking or overexpressing SHP2 were generated to examine the expression of PD-L1 and viral replication after WSN or PR8 infection. These results showed that the expressions of PD-L1 and NP in both mRNA and protein levels were significantly decreased after knockdown of *SHP2* (Figure 9 and Figure 10), whereas overexpression of *SHP2* exhibited the opposite effects (Figure 11 and Figure 12). These observations demonstrate that SHP2 can regulate the expression of PD-L1, which is required for IAV replication.

### 2.5. PD-L1 Regulates Activities of the ERK Signaling during IAV Infection

**Figure 13:**
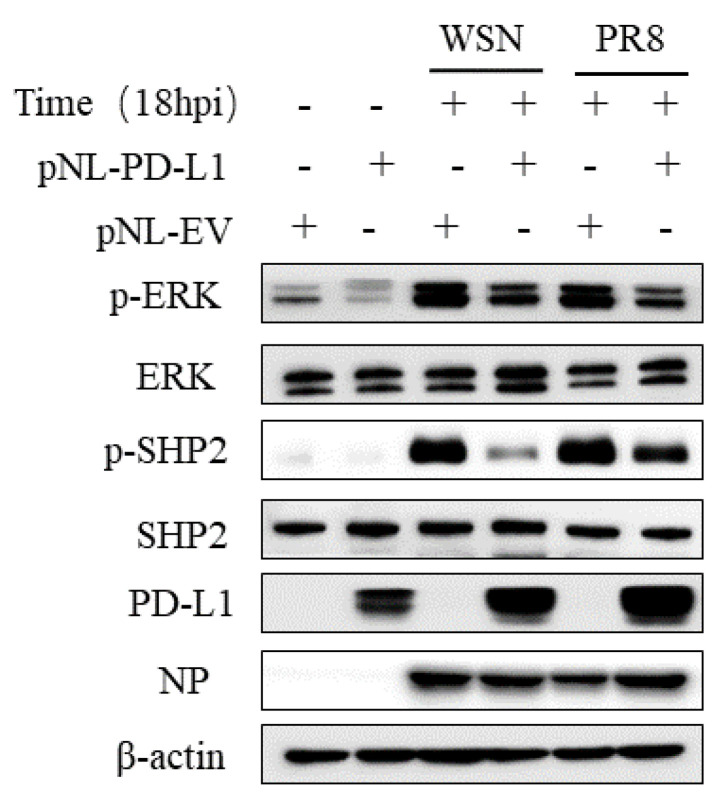
PD-L1 regulates the activation of ERK during IAV infection. A549 cells beading pNL-PD-L1 or pNL-EV were infected with or without WSN or PR8 (MOI = 1) for 18 h. The protein expression levels of ERK, p-ERK, SHP2, p-SHP2, and NP were detected by Western blotting. β-actin was used as internal standard. Three independent experiments were performed for each incubation.

Since SHP2 is able to mediate the PD-L1 expression, as well as the EGFR/ERK signaling, we further investigated the effect of PD-L1 on the EGFR/ERK signaling. For this reason, we examined the expression of p-ERK and p-SHP2 in A549 cells overexpressing PD-L1 following WSN or PR8 infection. The results showed that overexpression of PD-L1 led to the decreased expression levels of p-SHP2 and p-ERK induced by WSN or PR8 infection (Figure 13). These findings suggest that PD-L1 can negatively regulate the activities of the ERK signaling pathway.

## 3. Discussion

Although there remain many challenges in current clinical trials, targeting PD-L1 holds promise for the development of immune modulators to treat pulmonary damages induced by virus infection. The discovery of these functions of PD-L1 in cancer progression inspires us to assess the expression levels of PD-L1 and its potentials in viral pathogenesis. To determine the role of PD-L1 in IAV infection, genetic and pharmacological approaches were utilized in this study. The present results identified that PD-L1 plays a key role in maintaining the immune tolerance during IAV/H1N1 infection.

PD-L1 has been identified as a biomarker of immune-checkpoint; the induction of PD-L1 could be significantly induced both in normal immune cells and tumor-infiltrating immune cells to evade host immune surveillances [17,18,25,29]. In addition, several studies have shown that PD-L1 mediates pyroptosis in tumor cells [17,27], and pyroptosis is critical for host macrophages against pathogenic microorganisms. IAV infection can regulate cell pyroptosis [4,9,10] and trigger pulmonary inflammatory responses [10], which mediate virus immune escape [4,12]; however, little is known about the function of PD-L1 in IAV infection. The expression of PD-L1 is generally regulated by numerous inflammatory factors [17,18,25,26]. In this study, the increased expressions of PD-L1 in both transcript and protein levels were observed in A549 cells after PR8 and WSN infection. The expression levels of IFN-β, IL-29, ISG15, and MxA were diminished in PD-L1-overexpressed cells during WSN or PR8 infection, determining that PD-L1 mediates immunosuppression by negatively regulating the expression of the cytokines induced by IAV infection. The results indicate that the high levels of PD-L1 expression could also also detected in pathogen-infected non-immune cells and imply that PD-L1 may play a potential role in the inflammatory response during IAV/H1N1 infection. Moreover, the expression of PD-L1 in A549 cells was highly associated with the activation of SHP2 upon IAV/H1N1 stimuli, which may be related to the development of diverse pulmonary diseases [30] and IAV infection [37]. Here, we found that the elevated expression of SHP2 can induce the expression of PD-L1 in IAV/H1N1-infected cells, identifying the regulatory effect of SHP2 on PD-L1.

ERK pathway was reported to be a potential target for antiviral strategy of SARS-CoV-2 [41,42,43]. IAV escapes from antiviral innate immunity by activating ERK signaling [35]. Whether the ERK pathway mediates the immunosuppression of PD-L1 during IAV infection remains unclear. The phosphorylation of ERK could be induced by SHP2 [35]. In this study, the expressions of PD-L1 and NP, as well as viral replication in A549 cells, were promoted in cells overexpressing SHP2. These results suggest that PD-L1 may be a downstream factor of SHP2 during IAV/H1N1 infection, and the upregulation of PD-L1 by SHP2 could facilitate virus replication and evasion from the host immune surveillance. Thus, it seems as though the ERK pathway could affect the function of PD-L1 during IAV infection. Based on these observations, the regulation of PD-L1 on the activities of ERK and SHP2 was further investigated. The expression of p-ERK and p-SHP2 were decreased by overexpression of PD-L1 during WSN or PR8 infection, suggesting that PD-L1 could negatively regulate the activity of ERK signaling and thus maintain the immune tolerance in response to IAV infection. However, these results cannot exclude that the existence of other signaling pathways could mediate the immunosuppressive role of PD-L1 upon WSN or PR8 challenge.

Because of the limited number of effective drugs and vaccines, there is an urgent need of a better understanding of the pathogenesis of IAV. This study determined that elevated PD-L1 expression may act as a predictive biomarker of IAV infection and exhibit a significant immunosuppressive function. Evidence has demonstrated the biological significance of PD-L1 in pulmonary fibrosis, suggesting the potential of targeting PD-L1 to control IAV/H1N1 infection in the future. However, there some challenges still remain, such as the lack of predictive and prognostic biomarkers and immunotherapy-related adverse effects. Therefore, understanding the molecular mechanism underlying the regulation of PD-L1 expression will provide a new insight to improve the efficacy of anti-PD-L1 therapy.

## 4. Materials and Methods

### 4.1. Cells, Virus Strains, Inhibitor, and Plasmids

293FT, A549, and MDCK cells were purchased from American Type Culture Collection (ATCC). All cells were cultured in Dulbecco’s modified Eagle’s medium (DMEM) supplemented with 10% (*v*/*v*) fetal bovine serum (FBS) (Gibco, Grand Island, NY, USA), 100 units of penicillin G, and 100 µg of streptomycin. All cells were cultured at 37 °C with 5% CO_2_ as previously described [44,45]. The culture medium was changed every day. The cells were passaged every three days with 0.05% trypsin-EDTA (25300-062, Gibco, USA) and seeded at a split ratio of 1:3. After three passages, cells were used for further experiments.

Influenza virus H1N1 subtype strain A/Puerto Rico/8/1934 (H1N1) (PR8), and A/WSN/22 (H1N1) (WSN) were propagated in specific-pathogen-free (SPF) chicken embryos as previously described [37,46,47]. Viruses were then harvested and preserved at −80 ℃. Virus titers were determined using a standard plaque assay in MDCK cells as described previously [38,44]. The titer of the virus stock was 6.5 × 10^6^ PFU/mL.

SHP099 was purchased from SELLECK (S8278, Houston, TX, USA).

For ectopic expression, the total RNA acquired from cells with a total RNA kit (Omega Bio-tek, Shenzhen, China) was reverse transcribed into cDNA using Rever Tra Ace qPCR RT master mix (Q712-02, Vazyme, Nanjing, China), and the coding regions of the SHP2 and PD-L1, according to the recently updated sequence available in NCBI GenBank (accession No. NM_002834.5, NM_0012 67706.2), were amplified by PCR. The amplicon was cloned into the eukaryotic expression vector pNL-EGFP/CMV/WPREdU3 at the Nhe I and Xho I cloning sites. Amplification primers for SHP2 and PD-L1 were designed by NCBI and synthesized by Shang ya Company, as shown in Table 1.

### 4.2. RNA Interference

For all the experiments, A549 cell lines were transfected with the indicated siRNA at a final concentration of 20 nM using Lipofectamine RNAiMAX (Invitrogen, Carlsbad, CA, USA), according to the manufacturer’s instructions. SHP2 siRNA (No. CRH3904) and their scrambled control siRNAs (No. CSR1000) were purchased from Cohesion Biotechnology. PD-L1 siRNA and their scrambled control siRNAs were purchased from TSINGKE. Cells were transfected with siRNAs or scrambled controls using Lipofectamine 2000, according to the manufacturer’s instructions (Invitrogen, Carlsbad, CA, USA). The cells were maintained for 72 h after siRNA transfection. For determination of the effects of SHP2 and PD-L1 depletion on viral replication, A549 cells were infected with IAV/H1N1/PR8 strain or WSN strain at an MOI of 1 for 1 h in DMEM medium supplemented with 2% FBS and 0.5% PS. The infectious medium was removed prior to adding a fresh medium (DMEM 5% FBS, 0.5% PS) at indicated time points. Viral RNA and infectious particles were then quantified. To assess the knockdown efficiencies of siSHP2 and siPD-L1, RT-PCR, quantitative RT-PCR (qRT-PCR) and Western blotting (WB) were performed.

### 4.3. Generation of Stable Cell Lines

Cell lines overexpressing SHP2, PD-L1, or empty vector (EV) were generated as previously described [44]. The lentiviruses encoding the SHP2, PD-L1, or an EV were produced in 293T cells by co-transfecting with pNL-EGFP/CMV/WPREdU3-SHP2 (pNL-SHP2),pNL-EGFP/CMV/WPREdU3-PD-L1 (pNL-PD-L1), pNL-VSVG, and pNL-package. The supernatants containing the lentiviruses were collected at 36 h post-transfection. RT-PCR, qRT-PCR, and WB were performed to assess the overexpression efficiencies of SHP2 and PD-L1.

### 4.4. Virus Infection and Inhibitor Treatment

In vitro infection with WSN or PR8 was performed under biosafety levels (BSL-2) laboratory conditions. A549 cells, siSHP2 cells, siPD-L1 cells, pNL-SHP2 cells, and pNL-PD-L1 cells were seeded in 6-well plates at 37 °C in 5% CO_2_. When the cells reached approximately 80–90% confluence, the cells were infected with the viruses at a multiplicity of infection (MOI = 1) for 1 h at 37 °C under 5% CO_2_. After adsorption of 1 h, the supernatants were aspired, and then the cells were washed with phosphate-buffered saline (PBS) and cultured in a fresh medium. The cells were harvested at 8 h post-infection (hpi) for gene expression analysis by RT-PCR and qRT-PCR, and at 18 hpi for protein expression analysis by WB. The supernatants were harvested at 18 hpi for virus titration by plaque assay.

One hour after infection with the viruses, the cells were washed with PBS and cultured in DMEM containing 2 μg/mL trypsin for the indicated time. Then, the cells were incubated with 10 μM SHP099. The cells were harvested at 8 hpi for gene expression analysis by RT-PCR and qRT-PCR and at 18 hpi for protein expression analysis by WB.

### 4.5. RT-PCR and qRT-PCR

Total RNA was isolated from cells at indicated time points using Trizol (Omega Bio-tek, Shenzhen, China), according to the manufacturer’s instructions, and then reverse transcribed into cDNA with M-MLV Reverse Transcriptase (Promega, Fitchburg, WI, USA). The cDNA was analyzed by RT-PCR using rTaq DNA polymerase (Takara, Tokyo, Japan). Amplicons from the RT-PCR reactions were separated on a 1.5% agarose gel, which was stained with nucleic acid stain I (Roche Diagnostics, Mannheim, Germany), photographed, and analyzed using the Gene Tools Analysis Software (Syngene, Cambridge, UK). The cDNA was performed by qRT-PCR using a LightCycler 96 instrument (Roche). The qRT-PCR data were presented as normalized ratios, which were calculated using ΔΔCT method by LightCycler system and software (Roche, Basel, Switzerland). The human *glyceraldehyde-3-phosphate dehydrogenase* (*GAPDH*) was used as an internal standard. The primer sequences are listed in Table 1.

### 4.6. WB

Cell lysates were extracted from the samples harvested at 18 hpi. The lysates were mixed with a sample buffer (Solarbio, Beijing, China) followed by heat treatment at 95 °C for 5 min. The samples were separated by SDS-polyacrylamide gel electrophoresis (SDS-PAGE). Bands were detected with primary antibodies specific to SHP2 (3397T, Cell Signaling Technology, Danvers, MA, USA), phospho-SHP2 (Y542) (3751T, Cell Signaling Technology, Cambridge, MA, USA), PD-L1 (3751T, Cell Signaling Technology), ERK (4695T, Cell Signaling Technology, MA, USA), phospho-ERK (T202/Y204) (4370T, Cell Signaling Technology), β-actin (R019, Transgen biotech, China), and anti-H1N1-NP (generated in our laboratory) at 4 °C overnight prior to incubation with horseradish peroxidase-conjugated goat anti-mouse (125229, Jackson ImmunoResearch Laboratories, West Grove, PA, USA) or anti-rabbit IgG (131879, Jackson ImmunoResearch Laboratories, West Grove, PA, USA) incubation for 2 h. The blots were developed using the FluorChem M Imaging System (Protein Simple, San Jose, CA, USA). β-actin was used as a reference of internal standard.

### 4.7. Plaque Assay

WSN or PR8 titers were determined by plaque assay as described previously [37]. Briefly, the collected supernatants were serially diluted with DMEM and added to monolayers of MDCK cells at −80% confluence in a 6-well plate. After incubation for 1 h at 37 °C with 5% CO_2_, the supernatants were aspired, and the covering medium containing 5% agarose was then overlaid on the cells at 4 °C for 30 min. After coagulation of agarose, the maintenance medium was added to the plates, and the plates were further incubated for 3 days for plaque formation. Subsequently, the cells were fixed with 10% formalin overnight and stained with crystal violet (1% *w*/*v*) for 30 min. Then, plaques were counted and the plaque forming unit (PFU) was determined.

### 4.8. Statistical Analysis

Data are presented as means± SEM. Values were considered statically significant at *p* ≤ 0.05. Statistical analysis was performed by one-way ANOVA using SPSS 21.0 (Chicago, IL, USA).

## Figures and Tables

**Table 1:** Primers used for RT-PCR and qRT-PCR.

Gene Name	Sequence (5′–3′) of Primer
*GAPDH*-F	TGGGTGTGAACCATGAGAAGT
*GAPDH*-R	AAGGCCATGCCAGTGAGCTT
*NP*-F	TCAAACGTGGGATCAATG
*NP*-R	GTGCAGACCGTGCTAAAA
*PD-L1*-F	GTTGTGGATCCAGTCACCTCT
*PD-L1*-R	AATTGGTGGTGGTGGTCTTAC
*SHP2*-F	GATTTTGTTCTTTCTGTGCG
*SHP2*-R	TAAGGGGCTGCTTGAGTTGT
*IFN-β*-F	GCTACAACTTGCTTGGATTCC
*IFN-β*-R	TGTCCTTGAGGCAGTATTCA
*IL-29-F*	TGCTGGTGACTTTGGTGCTA
*IL-29-R*	TGTGGTGACAGAGATTTGAACC
*ISG-15*-F	GCAACGAATTCCAGGTGTCC
*ISG-15*-R	TTCGTCGCATTTGTCCACCA
*MxA*-F	GTGCATTGCAGAAGGTCAGA
*MxA*-R	CTGGTGATAGGCCATCAGGT
*PD-L1-Nhe I*	TTTAGTGAACCGTCAGATCCGCTAGCATGAGGATATTTGCTGTCTTT
*PD-L1*-*Xho I*	TTGTAATCCAGAGGTTGATTCTCGAGTTACGTCTCCTCCAAATGTGT
*SHP2*-*Nhe I*	GTTTAGTGAACCGTCAGATCCGCTAGCATGACATCGCGGAGATGGTTTCA
*SHP2*-*Xho I*	TTTGTAATCCAGAGGTTGATTCTCGAGTCATCTGAAACTTTTCTGCTGTT

## Data Availability

The materials in this study are commercially available.

## References

[1] Bai L., Zhao Y., Dong J., Liang S., Guo M., Liu X., Wang X., Huang Z., Sun X., Zhang Z. (2021). Coinfection with influenza A virus enhances SARS-CoV-2 infectivity. Cell Res..

[2] Schweitzer K.S., Crue T., Nall J.M., Foster D., Sajuthi S., Correll K.A., Nakamura M., Everman J.L., Downey G.P., Seibold M.A. (2021). Influenza virus infection increases ACE2 expression and shedding in human small airway epithelial cells. Eur. Respir. J..

[3] Lin C.Y., Shih M.C., Chang H.C., Lin K.J., Chen L.F., Huang S.W., Yang M.L., Ma S.K., Shiau A.L., Wang J.R. (2021). Influenza a virus NS1 resembles a TRAF3-interacting motif to target the RNA sensing-TRAF3-type I IFN axis and impair antiviral innate immunity. J. Biomed. Sci..

[4] Atkin-Smith G.K., Duan M., Chen W., Poon I.K.H. (2018). The induction and consequences of Influenza A virus-induced cell death. Cell Death Dis..

[5] Varghese P.M., Mukherjee S., Al-Mohanna F.A., Saleh S.M., Almajhdi F.N., Beirag N., Alkahtani S.H., Rajkumari R., Nal Rogier B., Sim R.B. (2021). Human Properdin Released by Infiltrating Neutrophils Can Modulate Influenza A Virus Infection. Front. Immunol..

[6] Downey J., Pernet E., Coulombe F., Divangahi M. (2018). Dissecting host cell death programs in the pathogenesis of influenza. Microbes Infect..

[7] Laghlali G., Lawlor K.E., Tate M.D. (2020). Die Another Way: Interplay between Influenza A Virus, Inflammation and Cell Death. Viruses.

[8] Atkin-Smith G.K., Duan M., Zanker D.J., Loh L., Nguyen T.H.O., Koutsakos M., Nguyen T., Jiang X., Carrera J., Phan T.K. (2020). Monocyte apoptotic bodies are vehicles for influenza A virus propagation. Commun. Biol..

[9] Zhang J., Liu J., Yuan Y., Huang F., Ma R., Luo B., Xi Z., Pan T., Liu B., Zhang Y. (2020). Two waves of pro-inflammatory factors are released during the influenza A virus (IAV)-driven pulmonary immunopathogenesis. PLoS Pathog..

[10] Pleschka S., Wolff T., Ehrhardt C., Hobom G., Planz O., Rapp U.R., Ludwig S. (2001). Influenza virus propagation is impaired by inhibition of the Raf/MEK/ERK signalling cascade. Nat. Cell Biol..

[11] Wang C., Liu H., Luo J., Chen L., Li M., Su W., Zhao N., Liu S., Xie L., Jia Y. (2017). HA Triggers the Switch from MEK1 SUMOylation to Phosphorylation of the ERK Pathway in Influenza A Virus-Infected Cells and Facilitates Its Infection. Front. Cell. Infect. Microbiol..

[12] Botwina P., Owczarek K., Rajfur Z., Ochman M., Urlik M., Nowakowska M., Szczubiałka K., Pyrc K. (2020). Berberine Hampers Influenza A Replication through Inhibition of MAPK/ERK Pathway. Viruses.

[13] Ramos I., Smith G., Ruf-Zamojski F., Martínez-Romero C., Fribourg M., Carbajal E.A., Hartmann B.M., Nair V.D., Marjanovic N., Monteagudo P.L. (2019). Innate Immune Response to Influenza Virus at Single-Cell Resolution in Human Epithelial Cells Revealed Paracrine Induction of Interferon Lambda 1. J. Virol..

[14] Wang Q., Peng C., Yang M., Huang F., Duan X., Wang S., Cheng H., Yang H., Zhao H., Qin Q. (2021). Single-cell RNA-seq landscape midbrain cell responses to red spotted grouper nervous necrosis virus infection. PLoS Pathog..

[15] Julkunen I., Sareneva T., Pirhonen J., Ronni T., Melén K., Matikainen S. (2001). Molecular pathogenesis of influenza A virus infection and virus-induced regulation of cytokine gene expression. Cytokine Growth Factor Rev..

[16] Chen J., Jiang C.C., Jin L., Zhang X.D. (2016). Regulation of PD-L1: A novel role of pro-survival signalling in cancer. Ann. Oncol..

[17] Keir M.E., Butte M.J., Freeman G.J., Sharpe A.H. (2008). PD-1 and its ligands in tolerance and immunity. Annu. Rev. Immunol..

[18] Benci J.L., Johnson L.R., Choa R., Xu Y., Qiu J., Zhou Z., Xu B., Ye D., Nathanson K.L., June C.H. (2019). Opposing Functions of Interferon Coordinate Adaptive and Innate Immune Responses to Cancer Immune Checkpoint Blockade. Cell.

[19] Peng S., Wang R., Zhang X., Ma Y., Zhong L., Li K., Nishiyama A., Arai S., Yano S., Wang W. (2019). EGFR-TKI resistance promotes immune escape in lung cancer via increased PD-L1 expression. Mol. Cancer.

[20] Herbst R.S., Baas P., Kim D.W., Felip E., Pérez-Gracia J.L., Han J.Y., Molina J., Kim J.H., Arvis C.D., Ahn M.J. (2016). Pembrolizumab versus docetaxel for previously treated, PD-L1-positive, advanced non-small-cell lung cancer (KEYNOTE-010): A randomised controlled trial. Lancet.

[21] Gainor J.F., Shaw A.T., Sequist L.V., Fu X., Azzoli C.G., Piotrowska Z., Huynh T.G., Zhao L., Fulton L., Schultz K.R. (2016). EGFR Mutations and ALK Rearrangements Are Associated with Low Response Rates to PD-1 Pathway Blockade in Non-Small Cell Lung Cancer: A Retrospective Analysis. Clin. Cancer Res..

[22] Ribas A., Wolchok J.D. (2018). Cancer immunotherapy using checkpoint blockade. Science.

[23] Kalbasi A., Ribas A. (2020). Tumour-intrinsic resistance to immune checkpoint blockade. Nature reviews. Immunology.

[24] Han Y., Liu D., Li L. (2020). PD-1/PD-L1 pathway: Current researches in cancer. Am. J. Cancer Res..

[25] Ge J., Wang J., Xiong F., Jiang X., Zhu K., Wang Y., Mo Y., Gong Z., Zhang S., He Y. (2021). Epstein-Barr Virus-Encoded Circular RNA CircBART2.2 Promotes Immune Escape of Nasopharyngeal Carcinoma by Regulating PD-L1. Cancer Res..

[26] Schlößer H.A., Drebber U., Kloth M., Thelen M., Rothschild S.I., Haase S., Garcia-Marquez M., Wennhold K., Berlth F., Urbanski A. (2015). Immune checkpoints programmed death 1 ligand 1 and cytotoxic T lymphocyte associated molecule 4 in gastric adenocarcinoma. Oncoimmunology.

[27] Sieviläinen M., Passador-Santos F., Almahmoudi R., Christopher S., Siponen M., Toppila-Salmi S., Salo T., Al-Samadi A. (2018). Immune checkpoints indoleamine 2,3-dioxygenase 1 and programmed death-ligand 1 in oral mucosal dysplasia. J. Oral Pathol. Med..

[28] Cristescu R., Mogg R., Ayers M., Albright A., Murphy E., Yearley J., Sher X., Liu X.Q., Lu H., Nebozhyn M. (2018). Pan-tumor genomic biomarkers for PD-1 checkpoint blockade-based immunotherapy. Science.

[29] Yokosuka T., Takamatsu M., Kobayashi-Imanishi W., Hashimoto-Tane A., Azuma M., Saito T. (2012). Programmed cell death 1 forms negative costimulatory microclusters that directly inhibit T cell receptor signaling by recruiting phosphatase SHP2. J. Exp. Med..

[30] Parry R.V., Chemnitz J.M., Frauwirth K.A., Lanfranco A.R., Braunstein I., Kobayashi S.V., Linsley P.S., Thompson C.B., Riley J.L. (2005). CTLA-4 and PD-1 receptors inhibit T-cell activation by distinct mechanisms. Mol. Cell. Biol..

[31] Chen S.H., Dominik P.K., Stanfield J., Ding S., Yang W., Kurd N., Llewellyn R., Heyen J., Wang C., Melton Z. (2021). Dual checkpoint blockade of CD47 and PD-L1 using an affinity-tuned bispecific antibody maximizes antitumor immunity. J. Immunother. Cancer.

[32] Shin D.S., Zaretsky J.M., Escuin-Ordinas H., Garcia-Diaz A., Hu-Lieskovan S., Kalbasi A., Grasso C.S., Hugo W., Sandoval S., Torrejon D.Y. (2017). Primary Resistance to PD-1 Blockade Mediated by JAK1/2 Mutations. Cancer Discov..

[33] Schwartz C., Schmidt V., Deinzer A., Hawerkamp H.C., Hams E., Bayerlein J., Röger O., Bailer M., Krautz C., El Gendy A. (2022). Innate PD-L1 limits T cell-mediated adipose tissue inflammation and ameliorates diet-induced obesity. Sci. Transl. Med..

[34] Bian Y., Lin T., Jakos T., Xiao X., Zhu J. (2022). The Generation of Dual-Targeting Fusion Protein PD-L1/CD47 for the Inhibition of Triple-Negative Breast Cancer. Biomedicines.

[35] Wang Q., Pan W., Wang S., Pan C., Ning H., Huang S., Chiu S.H., Chen J.L. (2021). Protein Tyrosine Phosphatase SHP2 Suppresses Host Innate Immunity against Influenza A Virus by Regulating EGFR-Mediated Signaling. J. Virol..

[36] Sun C., Mezzadra R., Schumacher T.N. (2018). Regulation and Function of the PD-L1 Checkpoint. Immunity.

[37] Pan Q., Zhao Z., Liao Y., Chiu S.H., Wang S., Chen B., Chen N., Chen Y., Chen J.L. (2019). Identification of an Interferon-Stimulated Long Noncoding RNA (LncRNA ISR) Involved in Regulation of Influenza A Virus Replication. Int. J. Mol. Sci..

[38] Ouyang J., Zhu X., Chen Y., Wei H., Chen Q., Chi X., Qi B., Zhang L., Zhao Y., Gao G.F. (2014). NRAV, a long noncoding RNA, modulates antiviral responses through suppression of interferon-stimulated gene transcription. Cell Host Microbe.

[39] Liu B., Guo H., Xu J., Qin T., Guo Q., Gu N., Zhang D., Qian W., Dai J., Hou S. (2018). Elimination of tumor by CD47/PD-L1 dual-targeting fusion protein that engages innate and adaptive immune responses. mAbs.

[40] Carosella E.D., Ploussard G., LeMaoult J., Desgrandchamps F. (2015). A Systematic Review of Immunotherapy in Urologic Cancer: Evolving Roles for Targeting of CTLA-4, PD-1/PD-L1, and HLA-G. Eur. Urol..

[41] Schreiber A., Viemann D., Schöning J., Schloer S., Mecate Zambrano A., Brunotte L., Faist A., Schöfbänker M., Hrincius E., Hoffmann H. (2022). The MEK1/2-inhibitor ATR-002 efficiently blocks SARS-CoV-2 propagation and alleviates pro-inflammatory cytokine/ chemokine responses. Cell Mol. Life Sci..

[42] Schreiber A., Ambrosy B., Planz O., Schloer S., Rescher U., Ludwig S. (2022). The MEK1/2 Inhibitor ATR-002 (Zapnometinib) Synergistically Potentiates the Antiviral Effect of Direct-Acting Anti-SARS-CoV-2 Drugs. Pharmaceutics.

[43] Faist A., Schloer S., Mecate-Zambrano A., Janowski J., Schreiber A., Boergeling Y., Conrad B.C., Kumar S., Toebben L., Schughart K. (2023). Inhibition of p38 signaling curtails the SARS-CoV-2 induced inflammatory response but retains the IFN-dependent antiviral defense of the lung epithelial barrier. Antivir. Res..

[44] Chen Y.N., LaMarche M.J., Chan H.M., Fekkes P., Garcia-Fortanet J., Acker M.G., Antonakos B., Chen C.H., Chen Z., Cooke V.G. (2016). Allosteric inhibition of SHP2 phosphatase inhibits cancers driven by receptor tyrosine kinases. Nature.

[45] Liu J.J., Li Y., Chen W.S., Liang Y., Wang G., Zong M., Kaneko K., Xu R., Karin M., Feng G.S. (2018). Shp2 deletion in hepatocytes suppresses hepatocarcinogenesis driven by oncogenic β-Catenin, PIK3CA and MET. J. Hepatol..

[46] Freeman G.J., Long A.J., Iwai Y., Bourque K., Chernova T., Nishimura H., Fitz L.J., Malenkovich N., Okazaki T., Byrne M.C. (2000). Engagement of the PD-1 immunoinhibitory receptor by a novel B7 family member leads to negative regulation of lymphocyte activation. J. Exp. Med..

[47] Zheng W., Li J., Wang S., Cao S., Jiang J., Chen C., Ding C., Qin C., Ye X., Gao G.F. (2015). Phosphorylation controls the nuclear-cytoplasmic shuttling of influenza A virus nucleoprotein. J. Virol..

